# An Intelligent Evaluation Algorithm for Pilot Flight Training Ability Based on Multimodal Information Fusion

**DOI:** 10.3390/s26072245

**Published:** 2026-04-04

**Authors:** Heming Zhang, Changyuan Wang, Pengbo Wang

**Affiliations:** 1 School of Opto-electronical Engineering, Xi’an Technological University, Xi’an 710021, China; xatu_zhangheming@163.com; 2 School of Mechatronic Engineering, Xi’an Technological University, Xi’an 710021, China

**Keywords:** pilot flight training, multimodal information fusion, flight training ability, OODA loop

## Abstract

Intelligent-assisted assessment of pilot flight training ability is a method of automating the evaluation of pilots’ flight skills using artificial intelligence. Currently, using AI to assist or replace human instructors in flight skill assessment has become a mainstream research direction in the field of intelligent aviation. Existing flight skill assessment methods suffer from limitations in data types and insufficient assessment accuracy. To address these issues, we evaluate and predict pilot performance in simulated flight missions based on physiological signals. Following the “OODA loop” theory, we established a multimodal dataset including pilot eye movement, electroencephalogram (EEG), electrocardiogram (ECG), electrodermal signaling (EDS), heart rate, respiration, and flight attitude data. This dataset records changes in physiological rhythms and flight behaviors during pilots’ flight training at different difficulty levels. To enhance the signal-to-noise ratio, we propose an enhanced wavelet fuzzy thresholding denoising algorithm utilizing LSTM optimization. We address the problem of isolated features across different time frames in multimodal data modeling by introducing a multi-feature fusion algorithm based on STFT. Furthermore, by combining a high-efficiency sub-attention mechanism with a Transformer network, we construct a multi-classification network for intelligent-assisted assessment of pilot flight training ability, further improving the output accuracy of each category. Experiments show that our designed algorithm can achieve a classification accuracy of up to 85% on the dataset (5-fold cross-validation), which meets the requirements for auxiliary assessment of flight capabilities.

## 1. Introduction

With the gradual optimization and innovation of aircraft-assisted driving and guidance equipment, the incidence of aviation accidents caused by pilot operational errors is rising. According to the Airbus “2018 Commercial Flight Accident Investigation Report”, between 1999 and 2018, nearly 60% of accidents resulting in aircraft destruction and fatalities occurred during takeoff, approach, and landing, of which more than 70% were caused by improper subjective operation of pilots [1]. This is mainly manifested in the pilots’ insufficient basic operation level, low operational proficiency, and insufficient enforcement of flight regulations. The investigators’ analysis of flight operation data (including normal flight operations, events, and accidents) shows that the trend of pilots’ operational errors when performing manual flights is increasing. Human factors have become an important cause of flight accidents. Poor human–computer interaction results mainly cause the flight crew’s erroneous decisions and operations. This phenomenon reflects the weakness of the aircraft cockpit in detecting pilot driving behavior.

In the first half of 2013, the Federal Aviation Administration of the United States issued a SAFO safety alert (Safety Alert for Operators) to operators [2]. Its theme is “Manual Flight Operation,” which aims to “encourage operators to improve the manual flight operation capabilities of pilots.” The Federal Aviation Administration of the United States believes that to meet the needs of safe flight, the knowledge and skills of manual flight operations must be maintained and improved. The safety alert further discusses that modern aircraft automatic flight systems are often used (for example, autopilots). Unfortunately, the continuous use of these systems is not conducive to improving and maintaining manual flight knowledge and skills. Automatic flight systems are valuable tools for pilots that can improve safety and reduce pilot workload, thereby obtaining more precise operations. However, the continuous use of automatic flight systems may lead to a decrease in pilot control capabilities. To improve pilots’ manual operation ability, many airlines organize regular refresher training for pilots in response to the above situation and to re-evaluate pilots’ flying ability and behavior during the training process. For example, The Federal Aviation Administration (FAA) of the United States stipulates that pilots must conduct regular proficiency checks, including annual flight and simulator checks every six months [3]. In addition, the FAA also requires pilots to conduct additional proficiency checks in specific circumstances (such as long periods of no flight). The European Aviation Safety Agency (EASA) requires pilots to conduct a flight check and a simulator check every year to ensure that pilots maintain a high level of operating skills. In addition, EASA also stipulates that pilots must regularly attend refresher courses to update and consolidate their flying skills [4]. With the increasing demand for pilot flight training ability testing, studying a high-precision and high-efficiency flight ability assessment method has become a current research hotspot.

**Figure 1:**
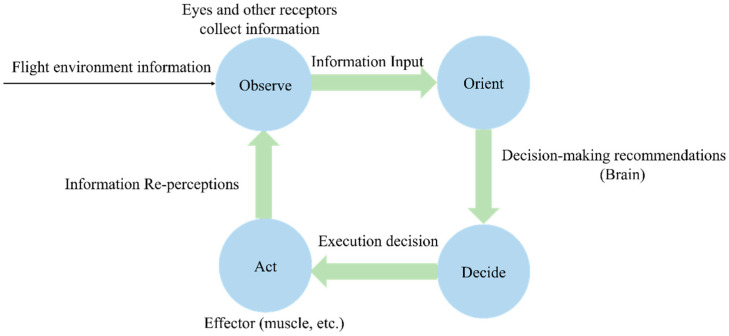
Diagram of the OODA loop.

Currently, the aviation industry’s control of pilots’ control behavior is based on the “OODA loop” theory [5]. The “OODA loop” theory is a closed-loop model proposed by Boyd in the 1970s, which is a cycle of four links: observation (Observe), cognition (Orient), decision (Decide), and action (Act). This theory can characterize all links in the pilot’s operation process, that is, the pilot forms cognition through observation and makes reasonable decisions to complete the action, and after the action, returns to the initial observation, thus forming a cyclical behavior process. The “OODA loop” can be used to comprehensively describe and evaluate the comprehensive capabilities of flight trainees in flight training. The schematic diagram of the “OODA loop” operation is shown in Figure 1.

Currently, the assessment standards for the standardization and proficiency of pilots’ flight behavior in the aviation industry are mainly based on the pilots’ flight performance. The assessment parameters of flight performance are mainly flight attitude, flight speed, and other data on the aircraft. The pilot’s operation is scored to examine the pilot’s control level. In the “OODA loop” theory, the flight attitude can only represent the pilot’s operation level in the “action” link. There is a lack of assessment of the pilot’s physiological behavior and psychological state during the “observation,” “judgment,” and “decision-making” process. Currently, flight instructors’ assessments of flight trainees’ observation, psychological, and decision-making abilities during flight training are relatively isolated, ultimately relying solely on flight attitude data for scoring. This approach, which uses only low-dimensional data, cannot adequately assess a pilot’s comprehensive capabilities. To improve the comprehensiveness of the assessment method, this paper links changes in cognitive alertness with pilot flight ability. The core hypothesis is that, in addition to traditional flight performance indicators, information contained in individual physiological responses can be used to detect the pilot’s cognitive state and predict flight performance. The affective computing and gaming literature suggest that when game difficulty aligns with a player’s ability, the player is in a state of “flow,” i.e., a state of active engagement and excitement [6]. When the difficulty does not match the player’s ability, the player may fall into a state of anxiety or boredom. Deviations from anxiety or boredom are associated with physiological changes, including alterations in heart rate, pupil dilation, respiratory rate, and skin conductance [7]. Therefore, the relationship between difficulty and ability can be assessed through physiological responses to evaluate pilot training capabilities.

To address the above problems in simulated flight ability assessment, we have conducted research on the following aspects:

(1) Existing automated flight capability assessment methods often focus only on flight parameters such as flight attitude and speed, neglecting the pilot’s physiological and psychological state during the “observation,” “judgment,” and “decision-making” processes. This paper, based on the “OODA loop” theory, collects pilot data including eye movements, EEG, ECG, skin conductance, heart rate, respiration, and flight attitude. Eye movement data is used to analyze the pilot’s observation behavior. ECG, skin conductance, heart rate, respiration, and flight attitude data are used to analyze changes in the pilot’s “action” phase. To better describe changes in the pilot’s cognitive alertness at each stage, this paper uses EEG data to characterize the pilot’s brain state at each stage. Through various biorhythms and control behavior data from pilots, this paper studies a scientific, comprehensive, and efficient method for assessing the control capabilities of flight trainees.
(2) Faced with a large amount of flight training data, traditional model training methods are inefficient and difficult to process and analyze data quickly. We mainly solve the problem of insufficient model training efficiency by solving the isolation of multimodal flight data.
(3) Existing assessment methods may not accurately reflect pilots’ comprehensive flight ability, especially their response ability in complex situations or emergencies. We will design an artificial intelligence network adapted to multimodal data to test pilots’ flight ability.

## 2. Related Work

In order to evaluate the safety of the driver’s control behavior and control status, researchers collect relevant data to characterize the driver’s behavior pattern in a particular aspect and evaluate the reliability of the driver’s driving behavior. The researchers mainly hope to test the driver’s single-aspect driving behavior ability through multiple data such as the driver’s driving data (such as direction, speed, acceleration, etc.), head and eye movement data, and EEG data.

### 2.1. Driving Behavior Assessment Method Based on Driving Data

Flight and driving data (such as direction, attitude, speed, acceleration, etc.) can most directly reflect the control behavior of the operator. This data reflects the behavior pattern of the driver in the “ACT” link to a certain extent and is currently the mainstream way to measure the control behavior ability of the operator. Researchers have conducted a series of studies on fully automatic control behavior evaluation based on the driving data of the aircraft or vehicle during driving. In order to solve the problem of flight safety assessment, Yang [8] proposed a bidirectional long short-term memory (Bi-LSTM) network prediction method combined with historical ADS-B data to establish the connection between control behavior and travel trajectory to achieve the purpose of controllability evaluation and safety warning. The experimental results show that Yang’s method has a high evaluation accuracy for the pilot’s driving behavior and also improves the aviation safety of the aircraft. In order to standardize and evaluate the pilot’s operational standardization during landing, Li et al. [9] used flight control data to perform safety detection on different operating behaviors. This method uses a time series to perform data mining to finally achieve the pilot’s landing behavior characteristics, construct an expression of pilot behavior, and finally realize the safety detection of aircraft control. Hu [10] established a driving behavior control model based on driving data (direction, speed, displacement, etc.) and designed a new abnormal driving detection model based on deep learning. This detection model first proposed a stacked sparse autoencoder model for model training. The model was trained in a greedy, layer-by-layer manner. Experiments show that the proposed scheme achieves excellent performance in abnormal driving detection compared with the state-of-the-art schemes.

### 2.2. Driving Behavior Assessment Method Based on Physiological Data

By analyzing EEG data, we can obtain the driver’s mental state and detect the driver’s cognitive state. When the driving ability cannot cope with complex driving situations, excessive cognitive load will cause the driver to make erroneous operations. Therefore, EEG data can reflect the driver’s “judgment” and “decision-making” behavioral abilities. Yiu [11] used an electroencephalogram (EEG) and NASA task load index to evaluate the pilot’s psychological state and subjectively evaluate the pilot’s psychological workload. The researchers divided the difficulty of training according to different workloads. The researchers used a Bayesian neural network (BNN) to identify potential mental behavioral load from the EEG power spectrum density. After verification, the classification model performed better than other baseline algorithms, with an accuracy of 66.5% and an F1 score of 61.4%. Wu [12] designed an adversarial Bayesian deep artificial intelligence network to solve the problem of control safety when the pilot is cognitively fatigued. This method uses batch normalization and data enhancement algorithms to enhance the features of EEG data. Finally, experiments have proved that this algorithm can effectively achieve high-accuracy detection of fatigue driving behavior. To detect pilots’ subjective intentions in controlling aircraft attitude, Zeng et al. [13] designed a spatial attention EEG network based on electroencephalogram (EEG) signals to identify pilot control intentions. This method incorporates receptive field attention mechanisms and spatial convolution to optimize the network. Experimental results show that this method achieves a 95% accuracy rate in three-class classification on a specific task.

Eye movement data can reflect a variety of information, such as the driver’s gaze direction, physiological and psychological state. Therefore, in order to obtain the pilot’s or driver’s operational ability in the “observation” and “judgment” links in the “OODA loop,” researchers have used eye-movement detection equipment or tracking equipment to study the driving evaluation method from the aspects of pilot or driver observation and gaze behavior. Lefrançois [14] analyzed the flight performance and eye movement characteristics of professional airline pilots in a full flight simulator. The researchers performed manual approach scenario training on 20 pilots. By tracking the eye movement characteristics of the pilots during landing, they counted and judged whether their gaze behavior on different instruments met the requirements. After experiments, this method can distinguish different patterns of pilots’ eye movement behavior during training. This experiment laid the foundation for the subsequent study of pilots’ operational ability through eye movement detection. The researchers subsequently evaluated the operating behavior of drivers through different eye movement data. Lounis [15] used eye data to study the visual information acquisition, gaze distraction, and gaze patterns of novices and pilots. The experimenters collected pilots’ eye movement data in manual landing mission scenarios. They used cosine KNN (K nearest neighbor)-based machine learning and transformation matrix to accurately classify pilots with different flying abilities. This study achieved a practical assessment of the pilot’s operating level through eye movement characteristics and also fully proved that eye movement behavior during flight training can fully reflect the operating ability of the flight crew.

### 2.3. Behavior Evaluation Method Based on Multiple Signals

Researchers used single-dimensional signals to detect and assess the relevant behaviors of test subjects, but the limitations of the data dimensions restricted the accuracy of the detection method to some extent. Therefore, more and more researchers have shifted their focus to the flight ability evaluation of multi-information fusion. Sun et al. [16] considered the limitations of previous pilot evaluation standards. This method scored each pilot’s ability and introduced the VENN model, ultimately achieving a more comprehensive measurement of the pilot’s flight ability. Wang et al. [17] used electroencephalography (EEG), electrocardiography (ECG), and eye-tracking data to detect pilot behavior. They classified different pilot behaviors using a support vector machine (SVM) model by calculating parameters such as standard deviation, root mean square of standard deviation, and average fixation duration. The results show that effective detection of pilot behavior can be achieved through multi-dimensional data analysis.

As the number of modalities expands, researchers have begun to study algorithm design and network structure optimization. To address the problem of multimodal joint learning where multimodal data deviates from its original distribution, Zhu et al. [18] proposed a multi-dimensional homogeneous coding space alignment (MHESA) method, which learns the homogeneous joint space of EEG and eye-tracking data through a multimodal joint spatial encoder. Experiments demonstrate that the MHESA method achieves more stable performance in multi-source data detection tasks. Wu et al. [19] introduced a modality-general and modality-specific (MGMS) learning model to address the problem of complementarity in emotional representations between different modalities. By utilizing EEG and facial expression fusion, this method addresses the challenge of detecting subjects’ facial behavior and emotional state. After testing, the classification accuracy of this method for four emotional states can reach 86.01%. This further improves the classification accuracy of the model for different types of data fusion. Rao et al. [20] first studied the relationship between the pilot’s multimodal physiological information and the pilot’s flight ability in 2022. The study collected and made public the dataset “CogPilot” through human tests conducted at the Massachusetts Institute of Technology’s Artificial Intelligence Office. The study required pilots to perform flight missions of varying difficulty and collected various physiological data and flight control postures of pilots when their flight performance was qualified. The training was then conducted using the dataset to establish a pilot flight ability classification model. The study of this dataset directly proved that multimodal fusion data, such as pilot physiological data and flight control data, can achieve classification and prediction of pilots’ different flight abilities.

In summary, the current technologies for pilot flight ability testing are developing toward multimodality and high efficiency. Integrating pilot physiological status and flight data through multiple sensors has become a hot topic for future research to achieve efficient and high-precision pilot flight ability assessment.

## 3. Methods

**Figure 2:**
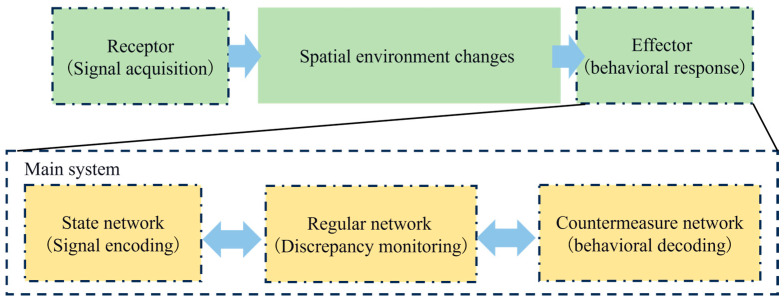
Pilot training control behavior patterns.

Holland summarized and refined a universal description framework for the human body’s adaptation to complex tasks, which includes four essential components: state, countermeasure (tree), and subject [21]. Individuals dominated by the nervous system are typical autonomous adaptive systems, so this framework can also describe them. Weiner pointed out that the nervous system is a circular process from the center to the effector, then to the sensory organ, and then back to the central nervous system [22]. Combined with Holland’s universal framework of complex adaptive systems, we can preliminarily outline the overall linkage architecture of the human nervous system for processing information. This shows that when different individuals deal with tasks of different complexity, their effectors will change according to their abilities. The structure includes four core components: the state network, the countermeasure network, the rule network, and the main structure composed of the three interconnected components. During flight training, the state network corresponds to the physiological and psychological state of the pilot during training. The countermeasure network represents the pilot’s operational behavior during the training process. The rule network represents a safe way of flying. Therefore, we propose to conduct a comprehensive assessment of the pilot’s flight ability based on the multimodal physiological data and flight control data generated by the effector during pilot training. The pilot training control behavior model is shown in Figure 2.

The blue arrow in Figure 2 represents the transmission of information. We continue to use the research conclusions of Rao et al. [20] and combine flight ability with flight changes in cognitive alertness. We evaluate the flight ability of different pilots by collecting data from pilots under different flight difficulty training. However, based on this, according to the “OODA loop” theory, during flight training, we require flight trainees to use their body receptors to capture flight deviations and changes in flight links during flight in different states through their eyes, then evaluate and judge the flight situation through the brain, and finally control the aircraft through the body to fly according to the prescribed behavior. In this process, eye movement data, EEG data, electromyography data, electrocardiogram data, and flight control data can be used to comprehensively measure whether the pilot’s various abilities (such as observation ability, positioning ability, cognitive ability, and control ability) can meet the safety flight requirements of the current flight mission difficulty.

**Figure 3:**
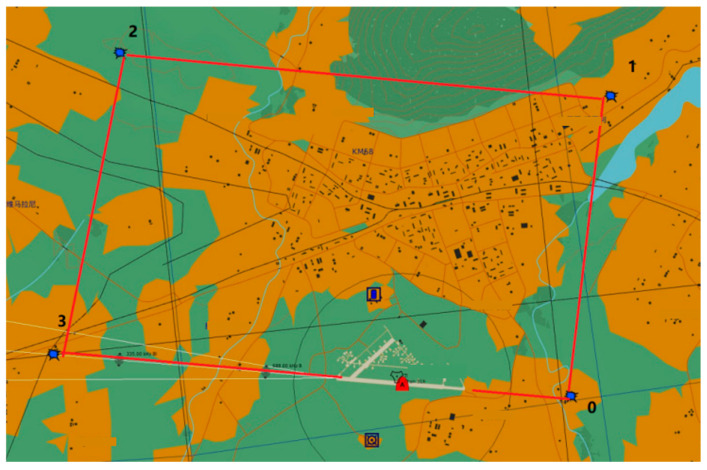
Flight track map.

In order to measure and evaluate the comprehensive flight ability of flight trainees, we reasonably divided the various flight tasks in flight training according to the total flight operation tasks (such as observation tasks, control tasks, average reaction time, etc.) in accordance with the “Civil Aviation Five-side Flight Operation Safety Regulations”, and evenly divided the training tasks of different difficulty values into four difficulty levels as data labels. In each flight training environment, we change the difficulty coefficient of the flight task by setting parameters such as wind speed, turbulence and visibility. For example, difficulty level 1 has no wind, excellent visibility, and cloud coverage above 2500 feet; difficulty level 2 simulates a crosswind from a 140° direction, a wind speed of 10 knots, and visibility of 5 miles; difficulty level 3 has light to moderate turbulence, cloud coverage above 1000 feet, and wind speed varies at different altitudes. In addition, the study also collected multimodal physiological data from the subjects, including electrocardiogram, respiration, acceleration, electromyography, skin electrodermal activity, photoplethysmography, eye tracking and head movement. These data were collected at a frequency of 512 Hz to 1024 Hz through sensors in different locations to evaluate the subjects’ performance throughout the flight. The flight trajectory diagram is shown in Figure 3.

The information of the data collection equipment is shown in Table 1. Based on the “OODA” loop theory, in order to measure the pilot’s “observation”, “judgment”, “analysis” and “decision-making” operational processes, we collected the pilot’s physiological data (such as: EEG, EMG, ECG and eye movement related data) and flight control data (flight attitude, flight speed, flight direction, altitude, descent rate) respectively, and conducted multimodal data monitoring of pilot behavior in different behavioral links.

We stored the collected multimodal data and difficulty labels to form a multimodal flight ability evaluation dataset. We constructed a multi-classification model between the pilot’s multimodal training data and flight difficulty through artificial intelligence network training. Through this model, we can compare the pilot’s predicted flight difficulty and actual flight difficulty in different flight environments based on the pilot’s multimodal data. Unlike previous studies, we aim to design and establish a set of efficient pilot training control ability multi-level classification models through efficient methods. This method can realize the multimodal automatic evaluation of pilot training behavior through low power consumption and stable evaluation results.

Based on the data distribution characteristics of flight ability detection using multimodal time series data, we use an innovative model architecture of frequency domain analysis and attention mechanism based on the improved data preprocessing method. We use frequency domain analysis to enhance the feature extraction of multimodal information and further optimize the utilization efficiency of features through the attention mechanism. Finally, the pilot’s flight ability level is output through the Transformer network.

### 3.1. Flight Training Multimodal Data Preprocessing

**Figure 4:**
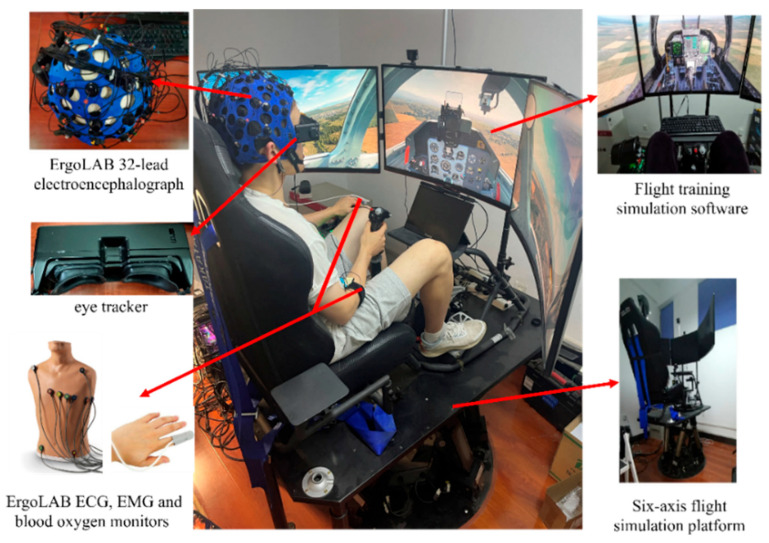
Flight training multimodal data collection process diagram.

To comprehensively evaluate pilots’ overall flight performance, this paper assesses their control behaviors during flight training. We collected physiological and flight control data from pilots during five-sided flight maneuvers, including eye movement, electroencephalogram (EEG), electrocardiogram (ECG), electrodermal signaling (EDS), heart rate, respiration, and flight attitude data. During data collection, subjects simulated flight training using the X-Plane 11 flight software on a T-6 Texan II aircraft. EEG signals were collected using the ErgoLAB 32-lead z EEG acquisition device to capture brain activity during the training process. EMG and EDS signals were obtained using ErgoLAB’s ECG, EMG, and pulse oximeters. Eye tracking and eye data (such as saccades, nystagmus, gaze placement, and eye acceleration) were collected using a vestibular function detector independently developed by the Artificial Intelligence Laboratory of Xi’an University of Technology. This device, utilizing artificial intelligence algorithms, can accurately capture multiple eye movement-related data points. The process diagram of multimodal data collection for flight training is shown in Figure 4.

#### 3.1.1. Wavelet-Adaptive Prefilter Based on LSTM Optimization

Since flight training requires simulated flights in different environments, multiple data, such as flight attitude, will be affected by noise interference. In order to avoid effective information being drowned in noise, we first pre-filtered the multimodal data. A pre-filter is a filter that pre-processes the signal before the data is output and used. It is often used to eliminate noise interference, enhance signal quality, etc., to ensure the accuracy and stability of the output signal. When selecting a pre-filter, it is necessary to comprehensively consider factors such as signal type, noise characteristics, system requirements, and implementation difficulty. Most of them use simple traditional filters to process signals. For example, the following common filter types include low-pass, high-pass, band-stop, and adaptive filters. When sensors work in harsh environments, they will be affected by environmental uncertainties, especially jitter, displacement, etc., and the noise of multiple sensors themselves may cause many signals and noises to overlap. In order to improve the filtering efficiency of multimodal data, we propose a wavelet-adaptive pre-filter based on LSTM optimization to dynamically adjust the filter parameters according to the data distribution characteristics of different signals to meet the filtering needs of multimodal data.

(1) Wavelet threshold filtering

**Figure 5:**
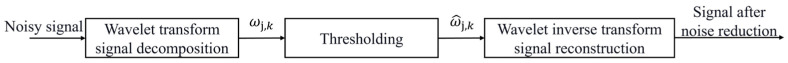
Wavelet threshold denoising process.

Wavelet threshold filtering was first proposed by Donoho. The wavelet threshold denoising method can effectively retain important information about the signal while filtering out noise and can adapt to the local characteristics of the signal. The algorithm is relatively simple and easy to implement. The process of the wavelet threshold denoising algorithm: First, select a suitable wavelet basis function to decompose the original signal to obtain a series of wavelet coefficients. Through the threshold and threshold function, the wavelet coefficients above the threshold are retained or appropriately shrunk and retained, and the wavelet coefficients below the threshold are zeroed. Finally, non-zero wavelet coefficients are selected to reconstruct the denoised signal. It can be seen that the selection of the wavelet basis function, decomposition layer number, wavelet threshold, and threshold function is crucial to the effect of wavelet denoising. Figure 5 shows the wavelet threshold denoising process.

In wavelet denoising, the hard threshold function has problems such as disconnection and discontinuity, leading to oscillation in the filtered signal. Although the soft threshold function is continuous, it has a fixed deviation, which will cause the problem of incomplete filtering. As for the selection of thresholds, the commonly used threshold estimation methods currently include fixed thresholds, maximum and minimum criterion thresholds, Stein unbiased likelihood thresholds, etc. Fixed thresholds are relatively simple but lack flexibility and have the problem of “over-killing.” The maximum and minimum criterion threshold has the problem of “over-retention” when the signal sample is large, and the unbiased likelihood threshold is unsuitable for processing low signal-to-noise ratio signals. The heuristic threshold also makes it easy to mistake useful high-frequency signals for noise and remove them. Therefore, based on the above analysis, this paper proposes introducing fuzzy logic by establishing a wavelet fuzzy threshold and threshold processing function based on two classic thresholds and fuzzy membership functions.

(2) Fuzzy Theory

Fuzzy theory is a method for dealing with fuzzy information and uncertainty problems. Unlike traditional binary logic, fuzzy theory allows things to exist between partially true and partially false, which aligns more with the uncertainty and fuzziness characteristics in the real world. The core idea of fuzzy theory is to introduce the concept of fuzzy sets, which have explicit binary attributes in traditional set theory. In fuzzy sets, each element can be represented by a certain membership degree, which can be any actual number between 0 and 1 to represent the degree of association with the set.

The main application areas of fuzzy theory include fuzzy control, fuzzy reasoning, fuzzy optimization, fuzzy decision-making, etc. Among them, fuzzy control is one of the most critical applications of fuzzy theory. In traditional control systems, precise mathematical models usually describe the relationship between input and output. In contrast, in fuzzy control, the uncertainty and fuzziness between input and output can be better handled, thereby achieving more flexible and robust control. In addition to control and reasoning, fuzzy theory can be applied to optimization problems. At present, fuzzy set theory has been widely used in linear programming. Therefore, this paper introduces fuzzy theory into the selection of threshold function and proposes an improved solution based on fuzzy threshold.

(3) Fuzzy threshold selection and fuzzy threshold function establishment

**Figure 6:**
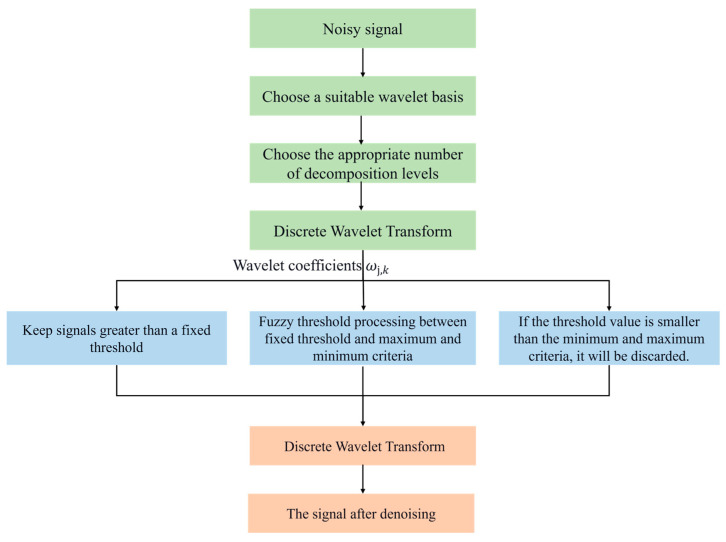
Improved wavelet fuzzy threshold method.

First, select the appropriate wavelet basis function and decomposition layer number, perform wavelet decomposition on the noisy signal to obtain a series of wavelet coefficients, remove the wavelet coefficients that are smaller than the maximum and minimum criterion threshold, retain the wavelet coefficients that are larger than the fixed value threshold, and process the wavelet coefficients between the maximum and minimum criterion threshold and the fixed threshold using the fuzzy membership function. Finally, an inverse transformation of the processed wavelet coefficients is performed to reconstruct the signal denoising. The specific improved algorithm is shown in Figure 6.

This paper uses the ascending semi-ridge fuzzy membership function to establish a wavelet fuzzy threshold denoising method that combines the threshold function with the threshold. The function expression is as follows:

(1) $$\mu_{A}(\omega_{j,k})=\left\{\begin{array}{cc} 0\;, & \omega_{j,k}<\lambda_{a}\\ \frac{1}{2}+\frac{1}{2}\sin\;(\frac{\pi (\omega_{j,k}-(\lambda_{a}+\lambda_{b})}{2(\lambda_{b}-\lambda_{a})}), & \lambda_{a}\leq \omega_{j,k}\leq \lambda_{b}\\ 1, & \omega_{j,k}>\lambda_{b} \end{array}\right.$$

(2) $$\begin{array}{c} {\overset{̑}{\omega }}_{j,k}=\omega_{j,k}\times \mu_{A}\left(\omega_{j,k}\right)=\\ \left\{\frac{1}{2}\omega_{j,k}\times (\begin{array}{cc} 0, & \omega_{j,k}<\lambda_{a}\\ 1+\sin\;(\frac{\pi (\omega_{j,k}-(\lambda_{a}+\lambda_{b})}{2(\lambda_{b}-\lambda_{a})})\;),\; & \lambda_{a}\leq \omega_{j,k}\leq \lambda_{b}\\ 1, & \omega_{j,k}>\lambda_{b} \end{array}\right. \end{array}$$

where $\lambda_{a}$ is the threshold of the minimax criterion, $\lambda_{b}$ is the fixed threshold, $\omega_{j,k}$ are the wavelet coefficients after decomposition, and $\mu_{A}\;(\omega_{j,k})$ is the fuzzy membership function.

(4) Layered adaptive wavelet fuzzy threshold denoising

Due to the difference in noise components in different frequency bands, this paper introduces a dynamic adjustment coefficient *r* based on the fuzzy threshold function. According to different decomposition scales, each layer selects the corresponding threshold and implements hierarchical adaptive improvement of the threshold to obtain a threshold *T(j)* suitable for each layer. The improved threshold calculation formula is:

(3) $$T_{a}(j)=r\cdot \lambda_{a}$$

(4) $$T_{b}(j)=r\cdot \lambda_{b}$$

where r represents the dynamic adjustment coefficient, $\lambda_{a}$ represents the maximum/minimum criterion threshold, and $\lambda_{b}$ represents the fixed threshold. T(j) represents the threshold for each layer.

In wavelet analysis, the wavelet coefficients with greater signal correlation are relatively large, and the wavelet coefficients with less noise correlation are smaller. Therefore, according to different decomposition scales, each layer selects a corresponding threshold to achieve dynamic changes in the layered threshold. Use the logarithmic function to construct a dynamic adjustment coefficient *r*:

(5) $$r=\left\{\begin{array}{cc} 1, & j<2\\ \frac{1}{\mathrm{lg}j}, & \;2\leq j\leq 5\\ \frac{1}{\mathrm{lg}(j+1)}, & j>5 \end{array}\right.$$

where *j* is the number of decomposition layers.

Therefore, the new threshold function is:

(6) $$\;{\overset{̑}{\omega }}_{j,k}=\omega_{j,k}\times \mu_{A}\left(\omega_{j,k}\right)=\;\left\{\begin{array}{cc} 0\;, & \omega_{j,k}<T_{a}\\ \omega_{j,k}(0.5+0.5\sin\;(\pi (\omega_{j,k}- & \\ (\lambda_{a}+\lambda_{b})/2)/(\lambda_{b}-\lambda_{a}))), & T_{a}\leq \omega_{j,k}\leq T_{b}\\ \omega_{j,k}, & \omega_{j,k}>T_{b} \end{array}\right.$$

where $\lambda_{a}$ is the threshold of the maximum/minimum criterion, $\lambda_{b}$ is the fixed threshold, $\omega_{j,k}$ are the wavelet coefficients after decomposition, and $\mu_{A}\;(\omega_{j,k})$ is the fuzzy membership function

(5) Improved wavelet threshold denoising algorithm based on LSTM optimization

Traditional wavelet thresholding denoising only processes high-frequency components, neglecting the low-frequency noise of the gyroscope. This paper proposes an improved scheme: LSTM is used to smooth the low-frequency components of the sensor after wavelet decomposition, preserving the original signal trend. After wavelet decomposition, the resulting low-frequency coefficients represent the approximate contour and main trend of the signal, and their changes exhibit time-dependent relationships. Changes in flight attitude and physiological signals can be reflected in the low-frequency coefficient sequence. Long short-term memory (LSTM) networks are specifically designed for modeling long-sequence time dependencies. They can memorize and utilize historical information to predict or generate the output at the current moment, making them suitable for learning smooth, continuous trend signals. Therefore, we use LSTM to fit the low-frequency wavelet coefficient sequence.

The LSTM network is responsible for fitting the overall wavelet coefficient value with the help of the wavelet coefficients before this moment. The characteristics of the wavelet coefficients are established through the input gate, forget gate, and output gate of the long short-term memory network. Finally, different weights are set for different long short-term memory network hidden layer units, and the fitting results are finally output after training. Assume that the long short-term memory network has a total of *m* neurons, of which the output value of the *x*-th neuron is $c_{x}$, the weight of the neuron is $v_{x}$, and the output value is $y_{t}$.

(7) $$v_{x}=\frac{exp\left(c_{x}\right)}{\sum_{i=1}^{m}c_{i}}$$

(8) $$y_{t}=\sum_{i=1}^{t}v_{i}c_{i}$$

where *m* represents the number of neurons in the long short-term memory network, *x* represents the neuron’s index, $c_{x}$ is the output value, $v_{x}$ is the neuron’s weight, and $y_{t}$ is the output value

Using this method, we have solved the problem of low adaptability of traditional wavelet transform filters to signals with different numerical distributions. Through this method, we pre-filter multimodal data to remove interference in the dataset.

In this algorithm, the LSTM network learns and predicts the optimal dynamic adjustment coefficient *r*. As shown in Equations (3) and (4), the theoretical initial value of *r* is given by the logarithmic function of the decomposition layer *j*. To further improve the adaptability of the threshold to different signal characteristics and noise levels, we introduce a lightweight LSTM network to fit the complex nonlinear relationship between the coefficient *r* and the statistical characteristics of the wavelet coefficients of the current decomposition layer, thereby generating adaptive optimal thresholds *T_a_(j)* and *T_b_(j)* for each wavelet decomposition layer. The input to the LSTM is the mean of the high-frequency wavelet coefficients *ω_j_* of the current decomposition layer *j*. The output is the optimized dynamic adjustment coefficient *r_opt_*.

The LSTM is trained in an unsupervised manner. The training objective is to minimize the reconstruction error between the denoised signal and the ideal reference signal. We use the mean square error (MSE) as the loss function and update the parameters of the LSTM weights and the threshold function simultaneously through backpropagation, enabling the system to automatically learn the optimal adjustment coefficient *r*.

The LSTM unit has 32 hidden states. During training, we use a large number of noisy signals and their corresponding wavelet decomposition coefficients as samples. In each iteration, LSTM predicts *r_opt_* based on the input features, then calculates the threshold of this layer using Formulas (3) and (4), processes the wavelet coefficients and reconstructs the signal, and finally calculates the gradient and updates the network using the loss function.

#### 3.1.2. Multimodal Data Layer Fusion

According to aviation flight training regulations, formal five-side flight training includes multiple links such as takeoff, pull-up, descent, and landing, and its period and training subjects are relatively large. Therefore, the multimodal data generated during the training process has the characteristics of high dimension and large scale before being processed. In order to solve the problem of the complexity of the calculation amount brought by large-scale data, we need to rely on mighty computing power as a support to further integrate and extract features of the vast training data to achieve efficient evaluation of pilot operation ability. On the other hand, the research on multimodal data for pilot training evaluation only considers the frequency domain characteristics of a single physiological signal. It cannot capture the complementary relationship expressed by multiple physiological sensors at a certain moment during flight training, nor can it capture the frequency domain characteristics of multimodal data. In order to take both into account, we used a short-time Fourier transform to resample and fuse the data. In order to keep the data undistorted, it is necessary to combine physiological principles to ensure that each physiological information contains a complete physiological cycle. According to the timestamp, we intercepted the required eye movement, electrocardiogram, skin electricity, electromyography, and breathing data. We eliminated individual data that did not contain multimodal physiological information simultaneously.

To better analyze and process this data, we transform it into a time–frequency plot. This can be achieved using the short-time fast Fourier transform (STFT), a commonly used time–frequency analysis method. It decomposes a signal into two dimensions: time and frequency, and analyzes the changes in these dimensions. The multimodal dataset in this paper includes various signals such as eye movement (1024 Hz), electroencephalography (EEG) (256 Hz), electrocardiography/electromyography/electrodermal signals (1024 Hz), respiration (8 Hz), and flight attitude (256 Hz). Pilots performing structured flight training often have fixed and clearly defined task nodes, and their key physiological rhythms and behavioral patterns also have clear phases and specific data frequencies in time. STFT can stably map the signal within each window to the frequency domain, generating a standard two-dimensional time–frequency plot. Therefore, this method is suitable for analyzing quasi-stationary time series signals.

Furthermore, compared to wavelet transform, Hilbert–Huang, and other data fusion methods, STFT reduces the complexity and computational cost of data computation in the preprocessing stage. This method is better suited for processing large-scale, high-efficiency data from flight simulation training.

When STFT processes pilot multimodal data, it divides the original signal into several fixed-length windows. The signal within each window undergoes a Fourier transform to obtain the corresponding spectrum. The window length is typically fixed, ranging from tens to hundreds of milliseconds.

In this way, the spectral characteristics of the signal can be analyzed in each time window to obtain the frequency information of the signal changing over time. By moving the window, the time–frequency diagram of the entire signal is obtained.

The essence of the short-time Fourier transform is to divide the time domain signal on the time axis according to a certain window length and then perform the Fourier transform on each window after division to convert the one-dimensional time domain signal into a two-dimensional time–frequency domain matrix. Short-time Fourier transform is a time–frequency analysis method commonly used in acoustic signal research. Adobe Audition, Prat, and other acoustic signal processing and data labeling software are based on short-time Fourier transform. The calculation formula of the short-time Fourier transform is

(9) $$X_{STFT}(\tau ,f)=\int_{-\infty }^{\infty }x(t)h(t-\tau )e^{-j2\pi ft}dt$$

where x(t) is the time-domain signal at time *t*, *h* is the window function, and τ is the position of the center time of the window in the Fourier transform on the time axis.

**Figure 7:**
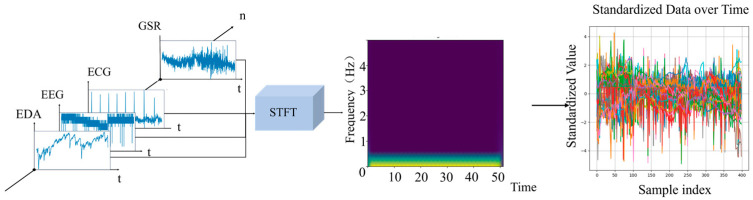
Multimodal data fusion flow chart.

In this paper, the Hamming window is used as the window function, the window function length is 0.5 s, and the window overlap rate is 50%. In order to ensure the consistency of the input data, the data is standardized. The multimodal data fusion flow chart is shown in Figure 7.

**Figure 8:**
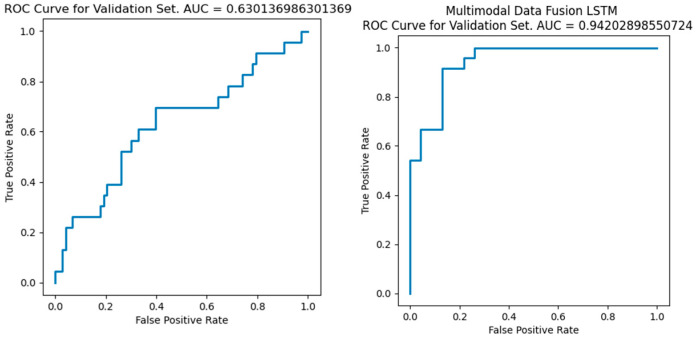
Test results of different fusion methods.

We performed LSTM prediction on the data after feature-layer fusion and the data without fusion and tested the performance of the network through AUC. As shown in the figure, it can be seen that the output results of the dataset after feature-layer fusion are significantly better than the related algorithms without fusion, and the AUC is greater than 0.94 and higher than the method without data fusion. The test results of different fusion methods are shown in Figure 8.

### 3.2. Transformer Pilot Operation Ability Evaluation Network Based on Efficient Multi-Scale Attention

To accurately and efficiently evaluate pilot training capabilities for multimodal data, we use an efficient multi-scale attention Transformer model to train the pilot’s comprehensive dataset. In this model, we use the EMA module to enhance the characteristics of the pilot’s comprehensive data and, at the same time, improve the classification accuracy of the model in multiple sensor data training tasks by combining it with the Transformer network, and ensure the pilot operation ability evaluation network maintains accurate output while maintaining low power consumption.

#### 3.2.1. Transformer Network Principle

**Figure 9:**
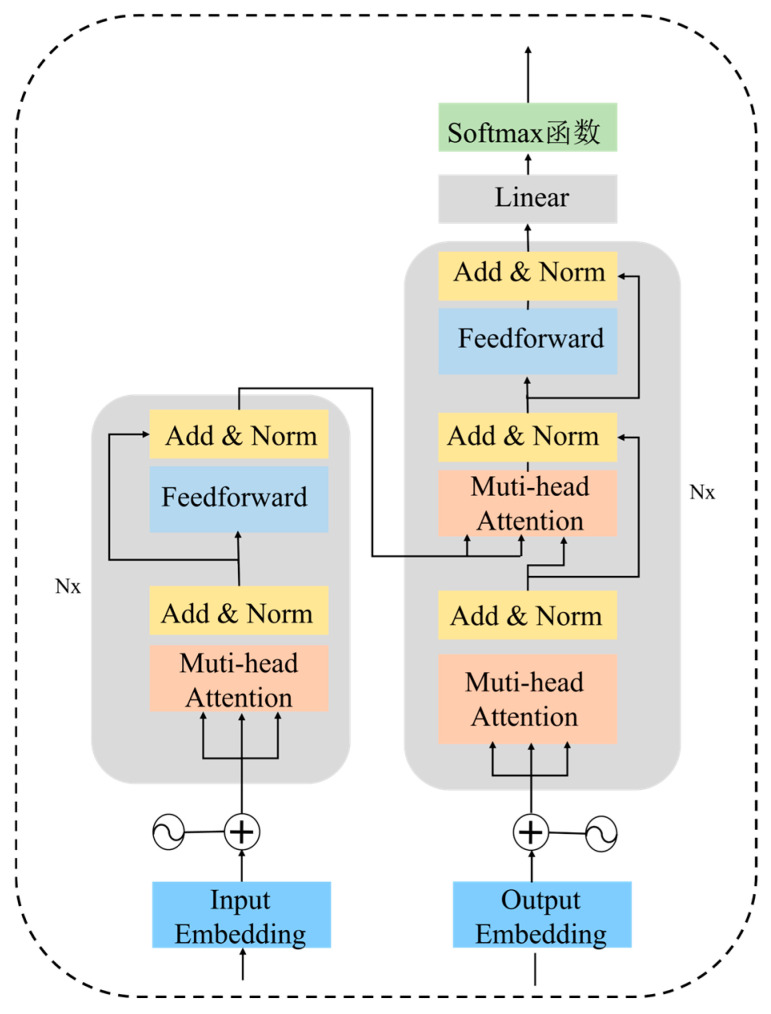
Transformer structure diagram.

The proposed network mainly uses the Transformer network to extract features from the pilot’s multimodal data. Transformer is a model with an attention mechanism. In recent years, it has demonstrated excellent performance in many fields and has gradually replaced existing CNN, RNN, and other models in sequence modeling problems. In contrast, the CNN structure makes it difficult to obtain global correlation when stacked in shallow layers, and the RNN structure makes it difficult to model long-distance dependencies of long sequences. The Transformer structure has better long-range dependency representation capabilities and can use the self-attention mechanism to capture local and global correlations of the input sequence. Transformer can be divided into two parts: encoder and decoder. Its structure is shown in Figure 9.

(1) Encoder

Each layer of the encoder consists of a multi-head attention layer (MSA), a feedforward connection layer (FF), a residual connection and a regularization layer (LN).

The multi-head attention layer contains multiple self-attention blocks with a set of learnable weight matrices, which can encapsulate the intricate relationships between elements in the vector. The self-attention mechanism reduces the influence of external information and focuses on the mining of internal correlations of data or features. It is a variant of the traditional attention mechanism.

Transformer’s self-attention mechanism uses scaled dot-product attention to calculate similarity, which saves more space than other attentions in calculation. Its calculation formula is:

(10) $$Attention(Q,K,V)=softmax\left(\frac{QK^{T}}{\sqrt{d}}\right)V$$

where Q is the query matrix. It represents the features that the attention mechanism searches for in the information based on specific task requirements. The attention mechanism weights the input sequence data according to the Q value at the current time. K is the key matrix. It represents a class of features of the input sequence obtained after computation. V is the value matrix. It represents the specific features within the input sequence. *d* is the dimension of Q, K, and V.

The main difference between the self-attention mechanism and the traditional attention mechanism is that the sources of query Q, key K, and value V are different. In the traditional attention mechanism, the values of Q, K, and V are defined according to specific purposes. In the self-attention mechanism, the values of Q, K, and V come from the input information. The calculation process of self-attention is shown in Figure 6.

First, the input feature vector is projected into new vectors, namely Q, K, and V. The mapping formula is:

(11) $$Q=F_{x}W^{Q}$$

(12) $$K=F_{x}W^{K}$$

(13) $$V=F_{x}W^{V}$$

where $F_{x}$ is the input feature vector; $W^{Q}$, $W^{K}$ and $W^{V}$ are the corresponding weight vectors.

Then, the similarity score between the input vectors is calculated, and the score value is $QK^{T}$. The similarity score can directly represent the degree of association between the elements at a certain position and the elements at other positions when encoding them. Next, the similarity score is normalized by dividing it by a scaling factor to ensure the stability of the gradient calculation. The scaling factor is $\sqrt{d}$. After that, the normalized similarity score is mapped to a probability value using the softmax function.

Finally, the mapped probability value is multiplied and accumulated with *V* to obtain the weight matrix, which is the output of self-attention. The vector with a larger weight receives more attention in the next layer.

(2) Decoder

In addition to the multi-head attention layer, feedforward connection layer, residual connection and regularization layer, the Transformer decoder also adds a masked multi-head attention layer (MMHA). The role of the masked multi-head attention layer is to mask all the subsequent data starting from the current position. This is because when the encoder is trained, the length of the data varies, so the decoder uses the maximum length of the data as the calculation unit when training. Its current calculation result is only affected by the previous data and is not affected by the subsequent data, so the subsequent data can be masked.

#### 3.2.2. Feature Layer Fusion Principle Based on EMA Module

This paper utilizes multimodal data, including eye-tracking, electroencephalography (EEG), electrocardiography (ECG), electromyography (EMG), electrodermal data, respiration, and flight attitude data. The spatial distribution characteristics of each modality differ. The time scales of physiological rhythm changes vary across different physiological data. The standard Transformer self-attention mechanism struggles to adaptively capture key dependencies at different scales when processing cross-modal and cross-scale multimodal data. Furthermore, to adapt to the efficiency requirements of pilot flight simulation training and reduce the computational complexity of auxiliary evaluation algorithms, this paper introduces an efficient multi-scale attention module (EMA) based on the Transformer model. This enhances the model’s feature extraction capabilities from multimodal data while reducing training complexity.

**Figure 10:**
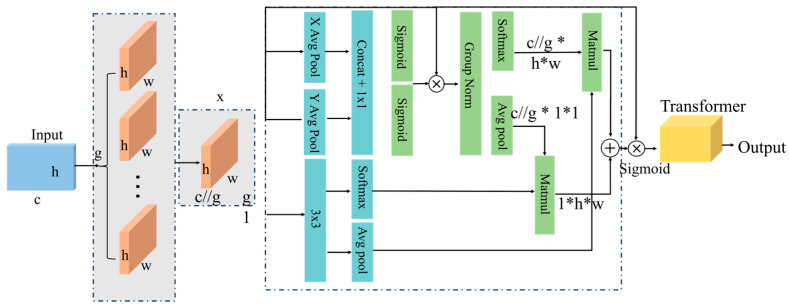
EMA module structure diagram.

The EMA module is an attention mechanism designed for processing sequential data. Compared to standard multi-head self-attention (MSA), this module, through a multi-branch parallel structure, channel recombination and grouping, and information feature fusion strategies, improves the module’s ability to simultaneously focus on local fine-grained features and global long-range trends. Compared to other attention modules (such as Linformer and Performer), the EMA (efficient multi-scale attention) module groups input features along the channel dimension. Each attention calculation is performed within only a subset, significantly reducing the scale of each matrix operation. Furthermore, EMA can further improve computational complexity and training efficiency by sharing weights across models. Therefore, this paper combines the Transformer with the EMA module. The Transformer network, when processing data with temporal progression characteristics, can model the long-range dependencies between any two time points in a sequence through a self-attention mechanism. EMA processes information at different scales in parallel through multiple branches and fuses them, improving the accuracy and training efficiency of the final classification task. The EMA module structure diagram is shown in Figure 10. h and w represent the dimensions of the extracted feature data, respectively.

#### 3.2.3. Loss Function

In order to adapt to the diverse data characteristics of pilot multimodal data, we use the multimodal orthogonalization loss function (*MMO* loss) to calculate the loss value of the network. *MMO* loss is a loss function used in multimodal learning that aims to encourage the model to extract complementary information from each modality. This loss function maximizes the information contribution of each modality to the final task by orthogonalizing the embedding representation of each modality, thereby improving the overall performance of the model. In multimodal time series data classification, *MMO* loss can ensure that the prediction error variance of the temporal context vector of different modalities can represent the amount of information on the temporal context of the modality for the classification task. Orthogonalization encourages the embedding of each modality learned by the model to be independent and complementary rather than interdependent or redundant. This helps the model better understand and classify multimodal time series data.

The calculation method of *MMO* loss usually calculates the difference between the nuclear norm of the sum of all modal embeddings and the sum of the nuclear norms of each modality embedding. The nuclear norm is the sum of the singular values of the matrix. By minimizing this difference, *MMO* loss penalizes those modes whose variance is reduced when merged. This loss is minimized when all unimodal embeddings are completely orthogonal. The specific mathematical expression is:

(14) $$L_{MMO}=\frac{1}{MN}\sum_{m=1}^{M}\max\left(1,∥∥h_{m}∥{∥}_{*}\right)-∥∥H∥{∥}_{*}$$

where *M* represents the number of modes, *N* represents the sequence length, *D* represents the embedding dimension, $h_{m}$ represents the embedding of the m-th mode, $H\in R^{l_{1}\times M\times N}$ is the set of embeddings of all modes, and $∥∥\cdot ∥{∥}_{*}$ represents a matrix.

This loss function is the difference between the sum of the nuclear norm of each embedding and the nuclear norm of all embeddings combined. It is penalized when the variance of the two modalities decreases separately but not when combined and is minimized when all unimodal embeddings are completely orthogonal. The norm of each modality is constrained to be at least 1 to prevent the embedding features from collapsing to zero. The design of this loss function enables the model to not only focus on the information extraction of a single modality during training, but also emphasizes the integration and complementarity of multimodal information, thereby improving the accuracy and efficiency of multimodal time series data classification.

## 4. Experimental Design and Analysis of Results

To test the performance of the multimodal pilot training capability detection model, we recorded the process of conducting five-sided flight simulation training tasks at four difficulty levels for flight trainees using the six-axis flight simulation training platform of the Artificial Intelligence Institute of Xi’an University of Technology. For experimental equipment and environment, we used the X-Plane 11 flight simulator for flight simulation testing. A high-performance workstation (Intel Xeon processor, 64 GB RAM) was used for model training and inference. TensorFlow 2.6 and PyTorch 1.9 were utilized to construct the network model and develop the experimental software.

During the experiment, we collected flight data from 25 flight trainees. Among the participating flight trainees, eight were novice flight trainees with less than 100 h of cumulative simulated flight training, eight had 100 to 200 h of simulated flight training, and seven had more than 200 h of simulated flight training. All flight trainees met the physical fitness requirements and health conditions stipulated by the Civil Aviation Administration of China. We equipped flight trainees with corresponding physiological monitoring devices and recorded various flight parameters. The sensors transmitted the data back to the host computer via a data bus, and the data for different difficulty levels for different trainees was stored in different databases. Each training flight departed from the same point, and the trainees had to use the instruments on the cockpit dashboard to guide the aircraft along an ideal path to the runway. This paper draws on the experimental paradigm of the U.S. Air Force Research Laboratory to set the difficulty of flight simulation training, dividing the difficulty of flight simulation training into four levels [19]. The difficulty of flight simulation training was adjusted by changing wind speed, turbulence, and visibility.

Flight difficulty classification table is shown in Table 2. Each participant was assigned a unique ID. Data was organized by participant ID, experiment date, and run number. The original time series data was stored in each run folder, with the file naming format as follows: sub-cpXXX_ses-YYYYMMDD_level-XXX_run-XXX_dat.csv. Here, “sub-cpXXX” represents the participant number, and “level-XXX” indicates the task difficulty level. This study employed a 5-fold cross-validation strategy. The experiment randomly divided the 25 pilot participants into five groups. In each round, data from one group of pilot trainees was selected as the test set and data from the remaining groups were used as the training set. In the experiment, each complete ILS task run for each subject was considered an indivisible data block. During partitioning, all time window samples from the same run must simultaneously enter either the training or test set. This scheme ensures complete isolation between the pilots in the training and test sets and maintains a balanced distribution of cognitive patterns through flight experience stratification. These data were used to form Dataset I for network performance testing.

To verify the reliability of the algorithm, we used the CogPilot dataset [20] collected from pilots as Dataset II. Dataset II contains data from 35 subjects flying simulated T-6 Texan II aircraft. Each subject completed 12 simulated flights. The dataset collected data on the subjects’ electrocardiogram, respiration, head posture, electromyography, electrodermal conductance, and eye movement. Dataset II adjusts the difficulty of flight training by setting different meteorological parameters in the X-Plane 11 flight software.

Dataset III uses the WAUC dataset [23], which was proposed by the Albuquerque research team. The dataset collected data on electroencephalogram, electrocardiogram, respiration, electrodermal conductance, and posture. The dataset collects multimodal data on the subjects during their driving behavior and classifies the subjects’ driving level. The dataset is classified by the number of driving tasks, and a multimodal driving dataset is constructed.

**Figure 11:**
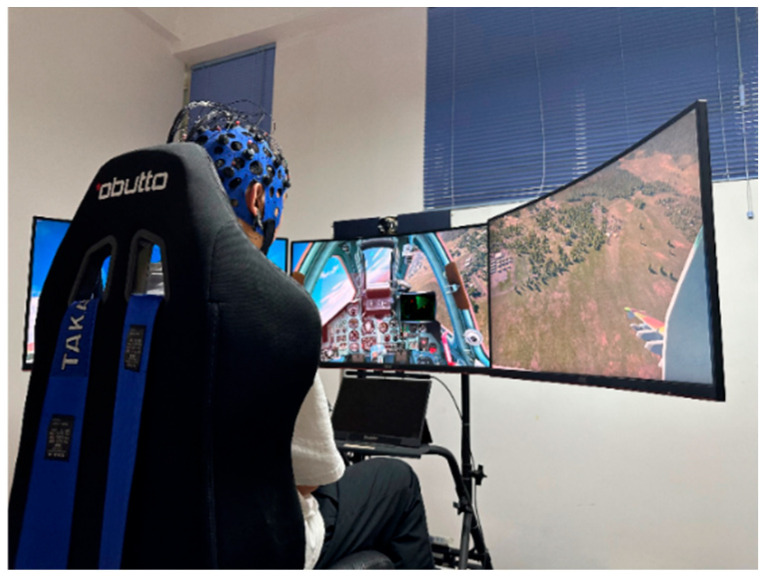
Pilot student vestibular function testing process diagram.

The pilot student vestibular function testing process diagram is shown in Figure 11. This paper proposes a Transformer-based pilot operational capability assessment network based on efficient multi-scale attention. The Transformer encoder has four stacked layers. Each layer has eight multi-head self-attention mechanisms, and the hidden layer dimension of the feedforward network (FFN) is 512. The EMA module has eight channel groups, and the multi-scale convolutional kernel sizes are 3, 5, and 7.

The model is trained using the Adam optimizer. The initial learning rate is set to 1 × 10^−4^. The batch size is 32. The weight decay coefficient is 1 × 10^−5^ to prevent overfitting. During training, a dropout layer with a dropout rate of 0.1 is used for regularization after the feedforward network layers. Training is monitored using the validation set loss as the metric. Early stopping is triggered when the loss does not decrease for 10 consecutive epochs, and the model parameters are restored to the lowest validation loss. In the experiments, each participant completes tasks at all four difficulty levels, thus ensuring a balanced class distribution in the original dataset. When performing 5-fold cross-validation splits by subject, we use a data management procedure to ensure that the number of class samples within each training fold and test fold remains balanced.

### 4.1. Prefilter Experiment

To verify the effect of the improved algorithm, we experimentally verified the denoising effect of the proposed LSTM-optimized wavelet-adaptive prefilter, and selected the optimal wavelet basis and decomposition layer number.

This paper uses the signal-to-noise ratio as the evaluation indicator for determining the number of decomposition layers. Usually, the number of wavelet decomposition layers of gyroscopes and accelerometers is three to eight. Using the above wavelet functions with relatively good effects, different wavelet decompositions are performed to obtain the corresponding signal-to-noise ratios. The experimental results are shown in Table 3.

From the signal-to-noise ratio of different wavelet basis functions at different decomposition levels in Table 3, it can be seen that most wavelet decomposition levels have the best effect when they are five to six levels. Among them, rbio2.6 wavelet and sym7 wavelet have better effect when they are decomposed into six levels, with signal-to-noise ratios of 20.3098 and 19.3769, respectively. Therefore, this paper selects rbio2.6 wavelet with 6 decomposition levels.

**Figure 12:**
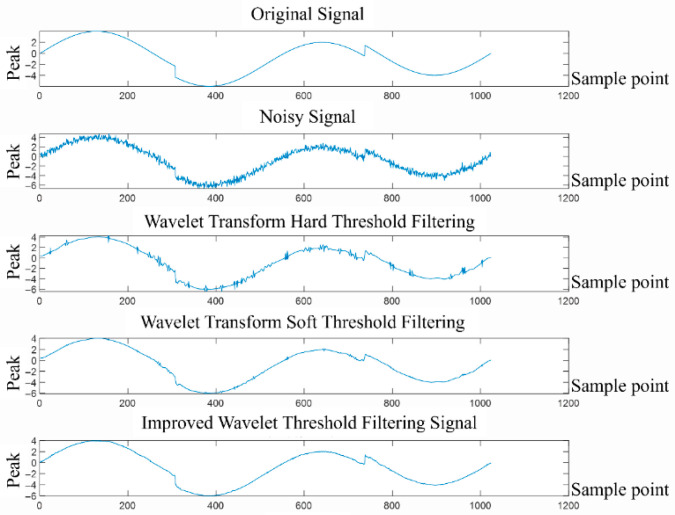
Comparison of processing results of different threshold functions.

We use different threshold functions for analysis and comparison. We select rbio2.6 wavelet with six decomposition levels. We use hard threshold, soft threshold and improved threshold for experimental simulation. The experimental results of different thresholds are shown in Figure 12, and the evaluation indicators after processing are shown in Table 3.

From the experimental results in Table 4, it can be seen that the evaluation coefficient of the improved threshold is 43.95% higher than the hard threshold and 11.02% higher than the soft threshold, and the mean square error is reduced by 58.01% and 15.08% compared with the hard threshold and the soft threshold.

**Figure 13:**
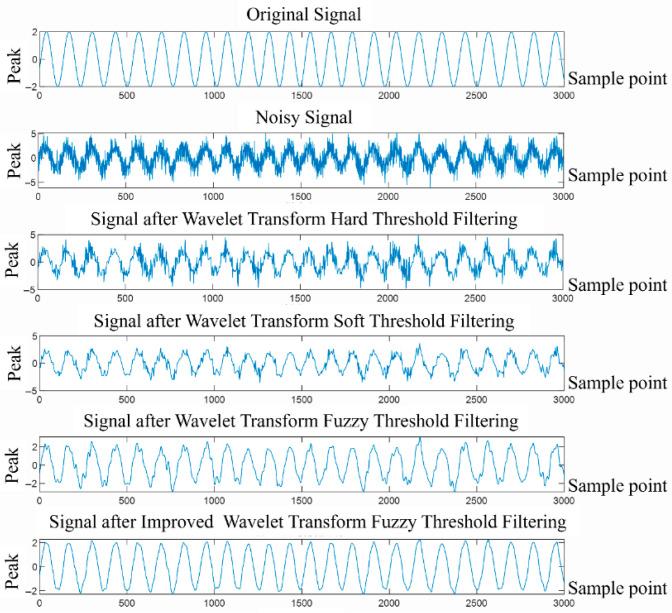
Results of different filtering methods for signals with a signal-to-noise ratio of 10 dB.

To further verify the superiority of the optimized wavelet denoising algorithm, different white noise sequences n(t) are added to obtain noisy signals with different signal-to-noise ratios: y(t)=x(t)+n(t), where $x(t)=2\sin(2\pi f_{1}t+\cos(2\pi f_{2}t))$. Different filtering methods are used to process it. The db6 wavelet is selected in the improved wavelet threshold algorithm after 5-scale decomposition, and experimental analysis is performed on it. As shown in Figure 13, the result of different filtering methods for signals with a signal-to-noise ratio of 10 dB is shown, and Table 5 shows the signal-to-noise ratio and root mean square of different schemes.

To verify the improvement effect of each module of the proposed LSTM-based adaptive wavelet fuzzy thresholding denoising algorithm on wavelet thresholding denoising, ablation experiments were conducted on each module. Hard thresholding, soft thresholding, wavelet fuzzy thresholding, least squares (LS) optimized adaptive wavelet thresholding, and the proposed LSTM-based adaptive wavelet fuzzy thresholding were used to process noisy signals with different signal-to-noise ratios. Table 5 shows that the fuzzy thresholding method outperforms the traditional hard and soft thresholding methods in filtering the four types of noisy signals. This indicates that the fuzzy thresholding method improves the flexibility of the filtering algorithm to a certain extent. Compared to the fuzzy thresholding method, the signal-to-noise ratio of the filtered signal after LS optimization of the adaptive wavelet fuzzy thresholding method is improved by an average of 3.7 dB. The average mean square error is reduced by 0.011. This result demonstrates that incorporating the hierarchical adaptive approach can improve the flexibility of the wavelet filtering algorithm and reduce the “over-suppression” of the real signal by the filtering algorithm. Compared to other methods, the LSTM-based adaptive wavelet ambiguity thresholding method proposed in this paper achieves the best filtering performance for four types of signals. The LSTM-based adaptive wavelet ambiguity thresholding method further improves the algorithmic flexibility compared to the least squares (LS) method. The mean square error of the filtered signals is lower than that of other methods. The mean square error of the filtered signal containing early noise reaches 0.1196, while the signal-to-noise ratio is 27.406. The validation results demonstrate that the LSTM-based adaptive wavelet ambiguity thresholding method proposed in this paper can effectively reduce signal noise while preserving the true signal.

### 4.2. Experimental Verification of Pilot Operational Ability Assessment Method

#### 4.2.1. Validation of Model Training Results

**Figure 14:**
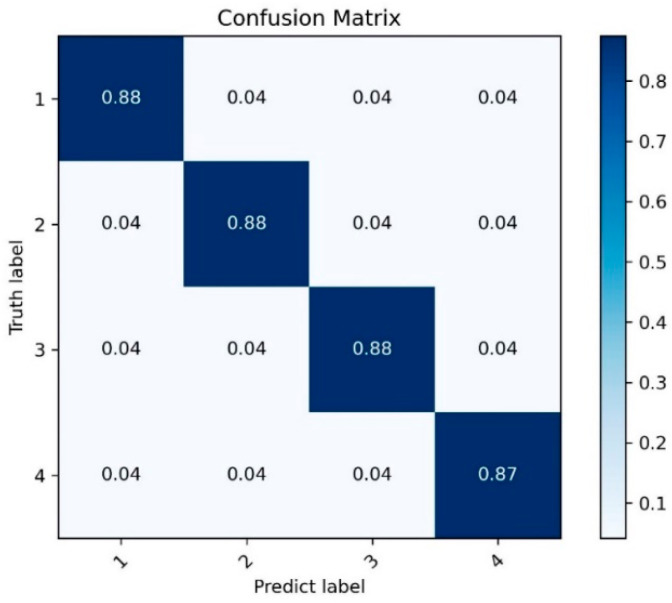
Confusion matrix of classification accuracy for each label.

We preprocessed the collected multimodal datasets, integrated the multimodal features into the flight capability classification network, and tested the classification results. We output the classification accuracy of each difficulty level label and the change of loss value during the training process. The loss value of our training decreased rapidly, and the classification accuracy of pilots at the four levels was slightly above 85%. Compared with previous studies, the training results of our network in Dataset I have been greatly improved. The classification accuracy of each label is shown in Figure 14.

In order to further test the classification accuracy of our proposed method for multimodal pilot datasets with different data distributions, we used the multimodal pilot driving ability detection algorithm to train Datasets I, II, and III, respectively and statistically analyzed the output results. In order to compare the improvement effect of our method over previous algorithms, we also trained baseline algorithms such as CNN, RNN, Transformer network, and a new, improved two-stream LSTM algorithm on Datasets I, II, and III. The new, improved two-stream LSTM algorithm is the latest algorithm proposed in the CogPilot Challenge. The algorithm uses a two-stream LSTM network to combine frequency domain features to classify flight multimodal data. In the CogPilot Challenge, the algorithm showed good classification results on the binary classification problem.

**Figure 15:**
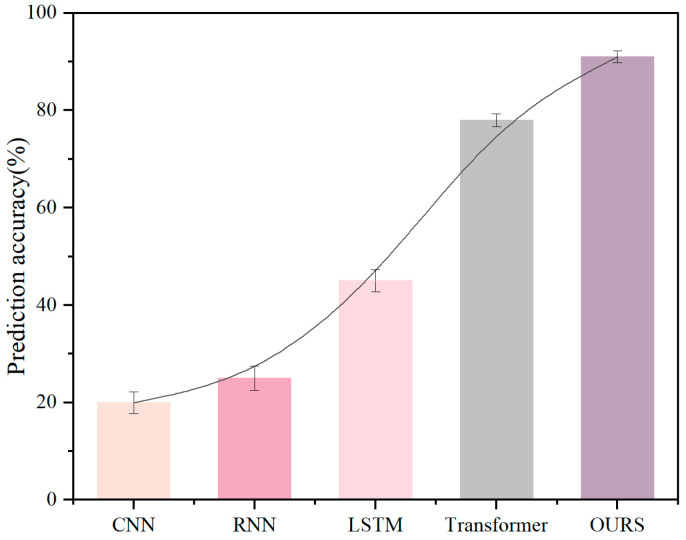
Comparison of training output results of Dataset I.

**Figure 16:**
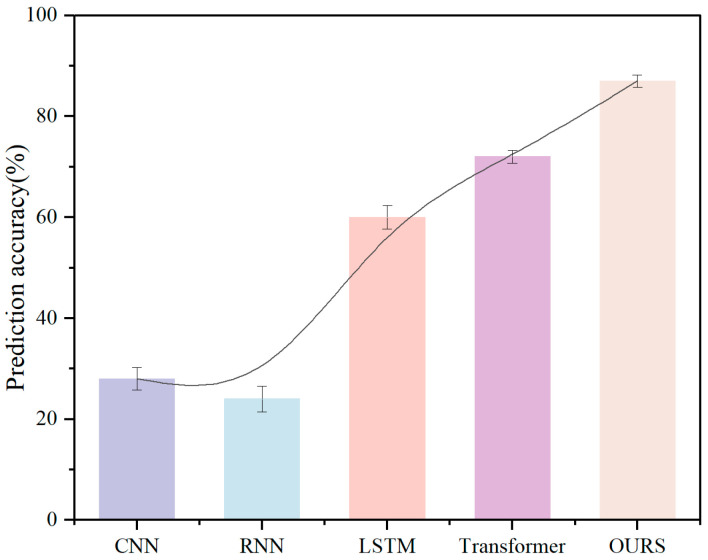
Comparison of training output results of Dataset II.

**Figure 17:**
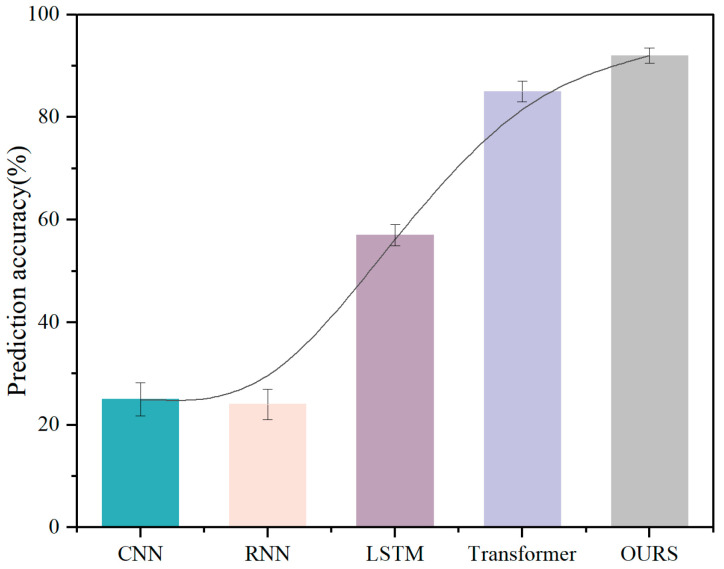
Comparison of training output results of Dataset III.

According to the training results of each network for different datasets in Figure 15, Figure 16 and Figure 17, we can see that the classification accuracy of the CNN network and RNN network for large batches and multi-dimensional data of different datasets is less than 30%, which cannot meet the requirements of pilot flight ability rating. Two-stream LSTM, Transformer, and our proposed method showed effective classification effects in the three datasets. First, for Dataset I, the classification rate of the two-stream LSTM network for four levels reached 47%. The Transformer network improved the algorithm accuracy by 12% compared with the two-stream LSTM network with its excellent data fitting and feature extraction capabilities. The classification method we proposed achieved an accuracy of 89% due to the Transformer module with a self-attention mechanism through the feature-layer fusion module. Secondly, as shown in Figure 16, after the test of Dataset II, we can see that due to the consideration of frequency domain signals, the classification accuracy of the improved LSTM network for the CogPilot dataset reached 65%. However, since the adjacent features between the multi-classification data and the binary classification data are not obvious enough, the two-stream LSTM network cannot achieve 90% accuracy for binary classification data. The algorithm we proposed not only takes frequency domain information into account but also improves accuracy by introducing pre-filtering and efficient self-attention mechanisms. The classification accuracy of our proposed classification method reaches 91% for the CogPilot dataset. Figure 17 shows that since the data dimension of Dataset III is lower than the previous two data dimensions, the classification accuracy of each dataset has improved. The classification rate of the Transformer network reached 84%. The classification rate of the network we proposed reached the highest, 93%. Therefore, in summary, the pilot flight ability assessment network of multimodal data we proposed can meet the needs of flight simulation training ability assessment.

To verify the reliability and statistical significance of the proposed multimodal flight capability assessment algorithm, we conducted a robustness evaluation of the model performance. The comparison of confidence intervals for results from different algorithms is shown in Table 6. Based on a 5-fold cross-validation strategy for model training and testing, we used Dataset I for training. We calculated the confidence intervals of the performance metrics for each algorithm across different data folds to measure the fluctuation range and stability of the algorithm’s prediction results.

The experimental results are shown in Table 6. Our proposed algorithm achieved the best robustness, with a confidence interval of (0.81 ± 0.04) for its performance metrics. This indicates that our proposed algorithm has a high mean classification accuracy with a small fluctuation range, demonstrating stable and reliable model performance. In contrast, the Transformer and RNN algorithms have wider confidence intervals and lower classification accuracy, indicating that their performance fluctuates significantly across different data subsets and lacks robustness. These experimental results demonstrate that our proposed algorithm achieves high classification accuracy while exhibiting superior statistical significance and generalization stability.

To verify the generalization ability of the proposed network model in cross-dataset scenarios, we designed a cross-dataset validation experiment. This paper uses Dataset I to train the model and tests and validates it on Dataset II. The experimental results are shown in Table 7. Our proposed EMA-Transformer model exhibits the best generalization performance, with accuracy, recall, and F1 score of 0.721 ± 0.032, 0.742 ± 0.023, and 0.742 ± 0.025, respectively, significantly outperforming baseline models such as standard Transformer, LSTM, and RNN. This result demonstrates that the features and patterns learned by the proposed method on Dataset I can be effectively transferred to datasets with different data distributions. The accuracy of cross-dataset validation is lower than the accuracy obtained from internal training and testing on Dataset II, but it reflects the robustness of the model when facing out-of-distribution data. This experiment shows that the proposed multimodal evaluation algorithm framework can capture the essential features of pilot cognitive load response that are common across datasets, verifying the good generalization ability and practicality of the method.

#### 4.2.2. Ablation Experiment Verification

In order to test the improvement effect of *MMO* loss function on training results, this paper uses cross-entropy loss, contrastive loss, and the *MMO* loss used in this paper to train the model to detect the impact of different loss functions on the training results of the model. The training results of contrastive loss, cross-entropy loss, and *MMO* loss are shown in the figure. From the table, it can be seen that *MMO* loss has a higher training accuracy for the network. As shown in the figure, *MMO* loss decreases the loss value faster than other loss functions during network training. The *MMO* loss function used in this article has a better fitting effect on multimodal data.

**Figure 18:**
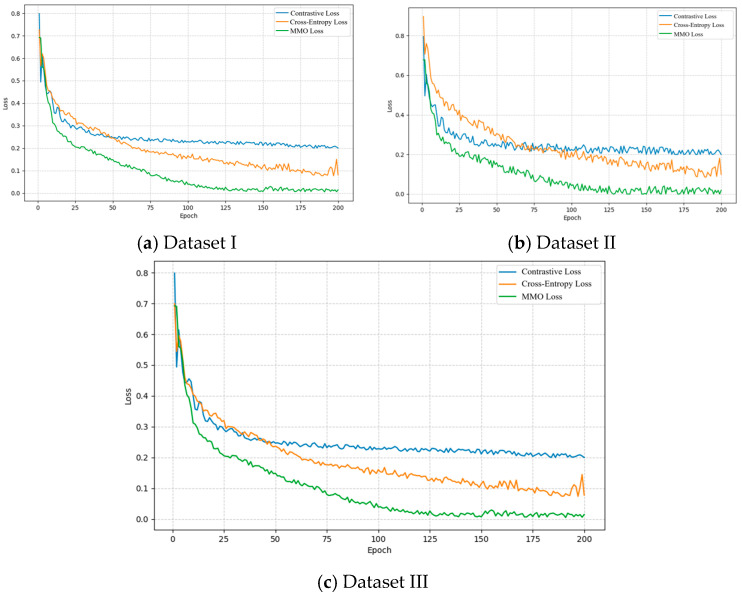
Comparison of loss curves with different loss functions.

According to Table 8, *MMO* loss exhibits significant advantages in model training. For the classification results of the three datasets, the model trained using *MMO* loss achieved a classification accuracy of 0.89, which is better than cross-entropy loss’s 0.87 and contrastive loss’s 0.80. In addition, the training process curve is shown in Figure 18, and *MMO* loss can cause the network loss value to decrease and converge at a faster rate. This confirms that *MMO* loss can effectively extract complementary information from multimodal data, effectively improving the model’s fitting ability and classification performance for complex multimodal temporal data.

To verify the performance improvement effect of the proposed efficient multi-scale attention (EMA) module on the model, we conducted ablation experiments on the “Transformer pilot operational ability evaluation network based on efficient multi-scale attention”. The experiment aims to compare and analyze the classification performance of the standard Transformer network, the Transformer network with integrated linear attention (Linformer), and our final proposed Transformer network with integrated EMA module (EMA Transformer) on three different datasets.

The experimental results are shown in Table 9, and the EMA module we introduced significantly enhances the model’s representational ability. On Dataset I, the classification accuracy of the standard Transformer network is 0.799 ± 0.024, while after integrating the EMA module, the accuracy is improved to 0.832 ± 0.023. On Datasets II and III, EMA Transformer also achieved optimal performance, with accuracies of 0.843 ± 0.014 and 0.851 ± 0.028, respectively, significantly higher than standard Transformer and Linformer + Transformer networks under the same conditions. In addition, EMA Transformer performs the best in recall and F1 Score metrics in most cases. This ablation experiment fully demonstrates that our proposed EMA module can effectively capture key dependencies across scales in multimodal temporal data, and construct a more efficient pilot operational capability evaluation model.

To verify the correctness of the fusion strategy proposed in this article, we classified the difficulty of flight training using different fusion methods. The results are shown in Table 10. Due to the inability to solve the feature fusion problem of multimodal data, the classification accuracy of using a single data-layer fusion method is only 0.34. Due to the feature isolation problem of simultaneous data, the classification accuracy of using a single feature-layer fusion method is 0.65. The method proposed in this article, which combines the data layer and feature layer, achieves a classification accuracy of 0.84 and a response time of 153 ms. It maintains a low response time while ensuring high classification accuracy. In order to test the classification performance of the fusion strategy in this article, we added a decision-level fusion method on the basis of the fusion strategy in this article for verification. As shown in the table, the addition of decision fusion improved classification accuracy by 0.5%, while the response time increased by 187 ms. This indicates that the improvement in classification method accuracy is limited after adding decision-layer algorithms, but it also increases response speed and computational complexity. A higher response time cannot meet the needs of pilot-assisted training.

## 5. Discussion

We studied the problems of incomplete standards, inefficient evaluation methods, and insufficient scientificity of evaluation methods in the current simulated flight training pilot’s comprehensive driving level evaluation process. We combined the research results of psychology and physiology on different people’s changes in cognitive alertnesss and studied an efficient evaluation method for simulated training flight ability based on multimodal training data. We collected multimodal data from pilots during flight training of different difficulty levels. We studied the mapping relationship between various physiological indicators of pilots and flight control data and pilot’s changes in cognitive alertness. According to each link of the OODA loop theory, we collected pilots’ multimodal physiological data (eye movement, EEG, ECG, skin electricity) and flight control data (heading, speed, descent ratio, etc.), respectively. We used training data and flight difficulty as data labels to study the efficient evaluation model of pilots’ multimodal flight ability.

In order to establish an accurate and efficient evaluation model of flight ability, we proposed a set of multimodal simulated flight ability detection methods. First, in view of the noise interference in multimodal data, we proposed an LSTM-optimized adaptive wavelet threshold denoising algorithm. Experiments have shown that the algorithm can effectively reduce the noise interference of different data. In addition, in order to solve the problems of large data scale and isolated features in the same period of flight data, we used short-time Fourier transform and the frequency domain information of the data to fuse the data at the same time, solving the problem of multiple data features being isolated from each other. The AUC value of the classification result after feature-layer fusion reached above 0.97. Finally, to achieve the best classification result and meet the requirements of flight ability evaluation, we designed and established a Transformer training network based on an efficient self-attention mechanism. We optimized the Transformer network with the help of an efficient self-attention mechanism. We used different data to verify the network. For different data sets, the prediction accuracy of the algorithm we proposed was above 85%, which was better than other algorithms. The classification accuracy of the CogPilot dataset was higher than the best classification result of the current CogPilot Challenge. In summary, the multimodal data flight ability evaluation method proposed in this paper realizes accurate, efficient, and comprehensive evaluation of the flight ability of different pilots.

## 6. Conclusions

This paper addresses the problems in the current pilot flight training capability assessment, such as single assessment dimensions, isolated multimodal data features, and insufficient model output accuracy. It proposes an intelligent auxiliary assessment algorithm for pilot flight training capability based on multimodal information fusion. First, based on the “OODA loop” theory, this paper constructs a multimodal assessment dataset that comprehensively reflects the entire chain of a pilot’s “observation–cognition–decision–action.” This dataset covers pilot eye movement, EEG, ECG, skin conductance, heart rate, respiration, and flight attitude data. Second, to improve data quality, this paper proposes an improved wavelet fuzzy thresholding denoising algorithm based on LSTM optimization, effectively suppressing multi-source noise. To address the multimodal data enhancement problem, this paper designs a data-layer fusion method based on the short-time Fourier transform. Finally, this paper combines an EMA module feature fusion module with a Transformer network to significantly improve assessment accuracy. Validation experiments on self-built and publicly available datasets demonstrate that the proposed algorithm framework achieves excellent performance in four-class classification tasks, with maximum accuracies of 89%, 91%, and 93%, respectively. Through ablation experiments, this paper demonstrates the improvement in classification accuracy achieved by each module. Future research will further expand the data scale and dimensions to include data from extremely complex situations and a broader group of pilots, thereby further enhancing the model’s generalization ability and robustness. On the other hand, future research will continue to explore more efficient network architectures or knowledge distillation techniques to achieve real-time, onboard pilot status monitoring. Looking ahead, the findings of this research can be applied to personalized training development and emergency response simulation training. This method can objectively quantify pilots’ decision-making and operational capabilities under pressure; furthermore, it can serve as an auxiliary tool for pilot selection and periodic retraining, providing multi-dimensional, data-driven capability assessment reports and offering new intelligent solutions for pilot training assessment.

## Figures and Tables

**Table 1:** Data acquisition equipment.

Device Name	Sensor Type	Collection Frequency
ErgoLAB EMG	EMG Sensor	1024 Hz
ErgoLAB HRV	HRV Sensor	8 Hz
ErgoLAB EDA	EDA Sensor	64 Hz
ErgoLAB ECG	ECG Sensor	1024 Hz
Xatu_Eyetracking	Eye Tracking Sensor	1024 Hz
ErgoLAB 32-lead	EEG Sensor	256 Hz
Xatu_III	Attitude sensor	256 Hz

**Table 2:** Flight difficulty classification table.

Training Difficulty Level	Visibility	Turbulence	Wind
1	Clear skies, unrestricted visibility	None	None
2	5 miles	None	140° @ 10 knots
3	3 miles	Light above 1000′	3000′: 80° @ 10 kts 1000′: 140° @ 10 kts
4	1 miles	Light	4800′: 250° @ 10 kts 2800′: 80° @ 15–20 kts 800′: 200° @ 15 kts

**Table 3:** Signal-to-noise ratio at different decomposition levels.

Wavelet Basis Functions	3	4	5	6	7	8
bior5.5	14.1931	16.6249	19.3811	20.1071	20.0301	16.9722
bior6.8	13.6216	16.2699	18.8757	19.2914	18.9834	18.0532
rbio3.7	12.7935	15.0150	16.0434	16.1259	15.3052	15.2589
rbio2.6	14.2115	16.6257	19.4999	20.3098	18.2401	17.5201
db6	13.2726	15.3182	16.4932	16.7248	16.7047	4.8769
sym7	13.6834	16.3456	19.0043	19.3769	18.9520	16.6071
coif3	13.6199	16.4100	18.3456	18.6848	18.6171	16.5672
sym5	13.7665	16.6183	18.6741	18.8736	18.5449	17.7607
sym8	13.9913	16.2847	18.8156	19.1479	18.8563	18.2189
db5	13.3444	15.9675	16.8837	17.1332	15.2156	14.4195

**Table 4:** Filtering effects of different threshold functions.

Index	Hard Threshold	Soft Threshold	Improved Threshold
SNR	14.1931	20.3098	21.7290
$\bar{SNR}$	43.3219	39.2877	40.7333
Mean square error	0.6022	0.2978	0.2529
Evaluation coefficient *σ*	6.14872	7.97925	8.85094

**Table 5:** Signal-to-noise ratio and root mean square of different schemes.

Noisy Signal		Hard Threshold Function	Soft Threshold Function	Wavelet Fuzzy Threshold	Adaptive Wavelet Fuzzy Thresholding Based on LS Optimization	LSTM-Based Adaptive Wavelet Fuzzy Thresholding
1 dB	SNR (dB)	5.7579	10.2193	11.4741	14.2178	16.8098
	MSE	0.7290	0.4362	0.4279	0.4128	0.4052
4 dB	SNR (dB)	9.1129	12.6740	13.8463	17.351	19.802
	MSE	0.4954	0.3288	0.3021	0.2833	0.2621
10 dB	SNR (dB)	14.3861	19.0948	20.7382	24.7250	25.8812
	MSE	0.2700	0.1570	0.1541	0.1459	0.1426
12 dB	SNR (dB)	17.6784	21.1366	22.2538	26.432	27.406
	MSE	0.1848	0.1241	0.1204	0.1201	0.1196

**Table 6:** Comparison of confidence intervals for results from different algorithms.

Classification Methods	Ours	Transformer	LSTM	RNN	CNN
Confidence interval	(0.81 ± 0.04)	(0.70 ± 0.13)	(0.61 ± 0.21)	(0.56 ± 0.21)	(0.50 ± 0.12)

**Table 7:** Comparison of accuracy for results from different algorithms.

Algorithm	Accuracy	Recall	F1-Score
RNN	0.229 ± 0.023	0.239 ± 0.045	0.219 ± 0.034
LSTM	0.350 ± 0.013	0.334 ± 0.024	0.346 ± 0.034
Transformer	0.525 ± 0.034	0.599 ± 0.031	0.599 ± 0.036
Ours	0.721 ± 0.032	0.742 ± 0.023	0.742 ± 0.025

**Table 8:** Comparison of training results with different loss functions.

Loss Function	Dataset I	Dataset II	Dataset III
Contrastive Loss	0.80	0.80	0.80
Cross-Entropy Loss	0.87	0.87	0.87
*MMO* Loss	0.89	0.89	0.89

**Table 9:** Ablation experiment results of different models.

Dataset	Algorithm	Accuracy	Recall	F1-Score
Dataset I	Transformer	0.799 ± 0.024	0.799 ± 0.035	0.799 ± 0.024
	Linformer + Transformer	0.819 ± 0.034	0.839 ± 0.075	0.839 ± 0.062
	EMA + Transformer	0.832 ± 0.023	0.842 ± 0.072	0.842 ± 0.06
Dataset II	Transformer	0.773 ± 0.042	0.799 ± 0.038	0.809 ± 0.057
	Linformer + Transformer	0.829 ± 0.036	0.829 ± 0.105	0.827 ± 0.077
	EMA + Transformer	0.843 ± 0.014	0.863 ± 0.046	0.863 ± 0.036
Dataset III	Transformer	0.783 ± 0.040	0.799 ± 0.034	0.799 ± 0.034
	Linformer + Transformer	0.846 ± 0.019	0.816 ± 0.046	0.816 ± 0.036
	EMA + Transformer	0.851 ± 0.028	0.841 ± 0.08	0.84 ± 0.06

**Table 10:** Training results of different fusion strategies.

Fusion Strategy	Accuracy	Response Time (ms)
Only data-layer fusion	0.34 ± 0.019	50
Only feature-layer fusion	0.65 ± 0.019	120
Data-layer + feature-layer fusion	0.846 ± 0.019	153
Data-layer + feature-layer + decision-layer fusion	0.851 ± 0.028	340

## Data Availability

The network training dataset and related program code used in this experiment have been uploaded to the github project. The data link is: https://github.com/617222535qqcom/Evaluate-and-test-the-flight-behavior-of-pilots-in-simulated-flight-training. (accessed on 1 May 2025). The DOI of the control dataset for this experiment is: https://doi.org/10.13026/tjpc-fm02. (accessed on 1 May 2025). The network link is: https://physionet.org/content/virtual-reality-piloting/1.0.0/. (accessed on 1 May 2025).
